# Postponed endoscopic necrosectomy results in a lower rate of additional intervention for infected walled-off necrosis

**DOI:** 10.1038/s41598-024-61675-2

**Published:** 2024-05-21

**Authors:** Songting Wu, Xiaotan Dou, Na Li, Hao Zhu, Lei Wang, Mingdong Liu, Chenggong Yu

**Affiliations:** Department of Gastroenterology, Nanjing Drum Tower Hospital, Affiliated Hospital of Medical School, Nanjing University, Nanjing, China

**Keywords:** Gastroenterology, Health care, Medical research

## Abstract

Although endoscopic necrosectomy (EN) is more frequently used to manage walled-off necrosis (WON), there is still debate over how much time should pass between the initial stent placement and the first necrosectomy. This study aims to determine the effect of performing EN within different timings after placing the initial stent on clinical outcomes for WON. A retrospective study on infected WON patients compared an early necrosectomy within one week after the initial stent placement with a necrosectomy that was postponed after a week. The primary outcomes compared the rate of clinical success and the need for additional intervention after EN to achieve WON resolution. 77 patients were divided into early and postponed necrosectomy groups. The complete resolution of WON within six months of follow-up was attained in 73.7% and 74.3% of patients in both the early and postponed groups. The early group tended to a greater need for additional intervention after EN (26.8% early necrosectomy vs. 8.3% postponed necrosectomy, P = 0.036). Our study does not demonstrate that early necrosectomy is superior to postponed necrosectomy in terms of clinical success rate, total count of necrosectomy procedures, procedure-related complications, length of hospitalization and prognosis. Conversely, patients in the postponed group received fewer additional interventions.

## Introduction

One of the most common digestive system emergencies worldwide is acute pancreatitis (AP). Infected necrotizing pancreatitis (INP) can develop in approximately 20–30% of AP patients, and WON can appear late in patients with INP^1^. WON, which is the collection of (peri)pancreatic necrotic tissue, is secondary to INP according to the revised Atlanta classification, which can be sterile or infected^2^. Additionally, the indications for drainage of WON included digestive tract or biliary obstruction, refractory pain, ongoing organ failure, persistent unwellness or rapidly enlarging WON after optimal medical therapy^3^. And approximately 38% of patients require interventions^3^. The treatment of WON has undergone great changes from open necrosectomy to a minimally invasive step-up approach, and an endoscopic step-up approach consisting of endoscopic ultrasound (EUS)-guided transluminal drainage (ETD) and endoscopic necrosectomy (EN) was later introduced^4,5^.

Recent evidence suggests that the endoscopic step-up approach is favoured over the step-up minimally invasive surgical approach, with a lower rate of pancreatic fistulas and a shorter length of hospital stay^6,7^. A multicenter, randomized trial showed that 41% of patients achieved clinical success with WON with transmural drainage and placement of plastic or metal stents, without additional necrosectomy^8^. Meanwhile, EN is necessary when drainage with stents fails to improve the clinical response or if the lesion contains massive solid necrotic material^9^. However, the timing of performing EN after endoscopic transmural drainage with stents is still controversial. Timothy et al. discovered that performing direct endoscopic necrosectomy during the initial endoscopic drainage can achieve a higher resolution rate, a shorter length of hospitalization and a lower rate of recurrence compared with only ETD for WON^10^. Similarly, Linda et al. also found that EN performed concurrently with the initial stent placement can minimize the necessity for additional necrosectomy procedures^11^. Conversely, some experts prefer waiting to perform EN after one week of the initial stent placement because it may reduce the risk of stent-related AEs and prevent unnecessary interventions^12^.

Consequently, the objective of this retrospective study was to evaluate the effect of the interval between EN and the initial stent placement on the clinical outcome of WON. Comparing the clinical success rate and the need for additional intervention after EN to resolve WON between the two groups were the primary objectives. The secondary objectives were to compare the overall number of necrosectomy sessions, procedure-related complications, length of hospitalization and prognosis between the two groups.

## Materials and methods

### Patients

The endoscopy databases in Nanjing Drum Tower Hospital were queried for all patients who had been diagnosed with walled-off pancreatic necrosis by contrast-enhanced computed tomography scan (CE-CT), magnetic resonance imaging (MRI) or endoscopic ultrasound (EUS) between December 2017 and June 2022. All patients were administered intravenous antibiotics before and after the procedure. We empirically use carbapenems or third-generation cephalosporins, and subsequently adjust antimicrobial therapy based on the results of drug sensitivity tests.

The study only included patients with infected WON, which was documented by the presence of gas on CE-CT or positive culture of cystic fluid, and they received EN after drainage with stents and had a follow-up of 6 months. Before being referred for endoscopy at our center, patients who had received endoscopic management or surgery for fluid collection in other facilities were excluded. In addition, the other exclusion criteria of our study were as follows: (1) patients with pancreatic pseudocysts, (2) patients with WON secondary to non-acute pancreatitis causes such as chronic pancreatitis, tumors, trauma, or surgery of the pancreas, (3) patients managed by percutaneous catheter drainage (PCD) or EUS-guided transluminal drainage (ETD) alone to resolve WON successfully and did not require any further intervention, (4) patients performed ETD but did not place the stent.

Baseline characteristics were calculated for each patient. Several indicators reflecting the severity of illness were also recorded, including CT severity index (CTSI), bedside index of severity of acute pancreatitis (BISAP), maximum size in cross section of WON, white blood cell count [WBC, the normal range is (3.5–9.5) × 10^9^/L] and C-reactive protein (CRP, the normal range is < 8 mg/L). It should be pointed out that a CT had to be performed within seven days before the initial necrosectomy, and BISAP, WBC and CRP had to be performed within 48 h. The following procedural details were collected from the hospital records: route and total count of necrosectomy procedures, days from the first stent placement to the necrosectomy. 

### Procedure technique

**Figure 1:**
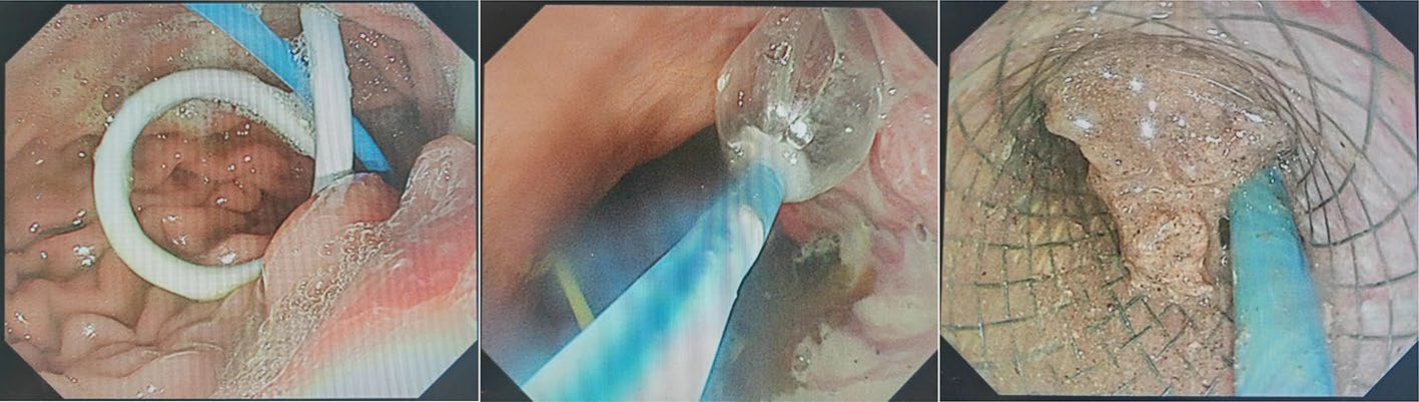
Endoscopic transmural necrosectomy (left: endoscopic insertion via the gastric wall; middle: balloon dilation of the puncture tract; right: the removal of necrotic tissue through the stent).

**Figure 2:**
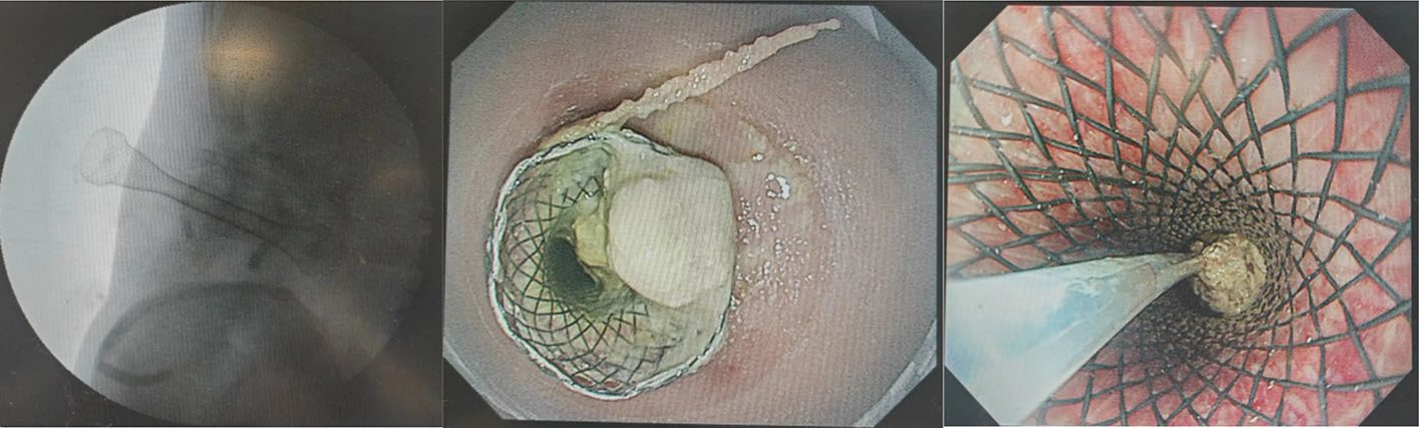
Percutaneous endoscopic necrosectomy (left: X-ray imaging of deployment of a covered self-expandable metal stent; middle: endoscopic insertion percutaneously; right: the removal of necrotic tissue through the stent).

To maintain the consistency of baseline data, all patients in our study underwent the initial invasive intervention after a disease duration of four weeks. Different intervention methods are selected based on the specific location of pancreatic necrosis as shown on enhanced CT scans. (1) Endoscopic transmural necrosectomy (ETN): All patients underwent endoscopic management with either general intravenous anaesthesia or tracheal intubation anaesthesia. EUS imaging was guided by Doppler flow to choose the best cyst puncture site, helping to prevent damage to mural blood vessels. The endoscopists used a 19-gauge needle for the puncture. After the guide wire was coiled into the WON, the physician withdrew the needle and sent the aspirate to the lab for microbiology. The cyst-enterostomy fistula tract was dilated using either an 8F–10F Soehendra Dilator or a 4 mm or 6 mm wire-guided balloon^13^. Following dilation, the endoscopists selected and inserted a double-pigtail plastic stent (DPPS), covered self-expandable metal stent (CSEMS) or lumen-apposing metal stent (LAMS). Following that, the stent was advanced along the guide wire, deploying the distal flange first and securing the WON wall under EUS guidance. The proximal flange was then released, tightly fitting the WON wall and the gastrointestinal tract^7^. If patients failed to demonstrate clinical improvement or if the necrotic collection persisted, ETN may be performed as needed. Subsequently, the adherent necrotic tissue was repeatedly separated with snares, baskets and rat-toothed forceps to prevent the distal flange from entangling and the stent from dislodging accidentally, and then any necrosis was removed into the gastric cavity^14^. In general, repeated operations were required until pink granulation tissue was observed. Then, endoscopists used normal saline to irrigate the necrotic cavity and observed active bleeding or not (Fig. 1). (2) Percutaneous endoscopic necrosectomy (PEN): All patients underwent percutaneous drainage (PCD) guided by X-ray, and after local infiltration anaesthesia, contrast agent was injected along the catheter, revealing irregular filling of the abscess cavity. The catheter was then removed, leaving the guidewire in place. Following this, a balloon dilator was inserted along the guidewire to dilate the puncture tract, and after balloon removal, a covered self-expandable metal stent (CSEMS) was placed^15^. If necessary, endoscopic necrosectomy was performed through the metal stent. This procedure involved passing a flexible endoscope accessories similar to those used in ETN to remove necrotic tissue (Fig. 2).

### Study outcomes

The primary study outcomes were comparing the clinical success rate of WON, which was defined as complete resolution of WON without recurrence and readmission at the six-month follow-up, and the additional intervention rate, which was defined as the need for additional intervention after EN during hospitalization, included PCD, ETD or surgery. The endoscopic reintervention performed during follow-up after WON resolution was not included in the calculation. The secondary outcomes were assessed by the following measures: (1) the overall number of necrosectomy sessions necessary to resolve WON; (2) the incidence of procedure-related adverse events, which included bleeding, septic shock, intestinal fistula, aspiration pneumonia and stent dislodgement; (3) the length of hospitalization; (4) the rate of WON recurrence; and (5) the incidence of long-term complications, which included pancreatic fistula, chronic pancreatitis, pancreatic portal hypertension, disconnected pancreatic duct syndrome (DPDS), intestinal fistula, and pancreatic exocrine insufficiency.

### Statistical analysis

Frequencies and percentages were used to express categorical variables, which were analysed by the chi-square test, Fisher exact or t-test. Continuous variables were summarized as the means and standard deviation (SD) (${\overline{\text{x}}}$ ± s) if they obeyed a normal distribution. Continuous data with a nonnormal distribution are summarized as medians and interquartile ranges [M (IQR)]. The Mann‒Whitney U test was used to evaluate continuous variables. The threshold for statistical significance was set a priori 0.05, and two-sided p values were used to compare all outcome measures. All statistical analyses were conducted with SPSS version 25 (IBM, United States).

### Informed consent

The consent is waived because it is a retrospective review study. Waiver for informed consent is approved by the Ethics Committee of Nanjing Drum Tower Hospital. All methods are carried out in accordance with relevant guidelines and regulations.

## Results

### Baseline characteristics

**Figure 3:**
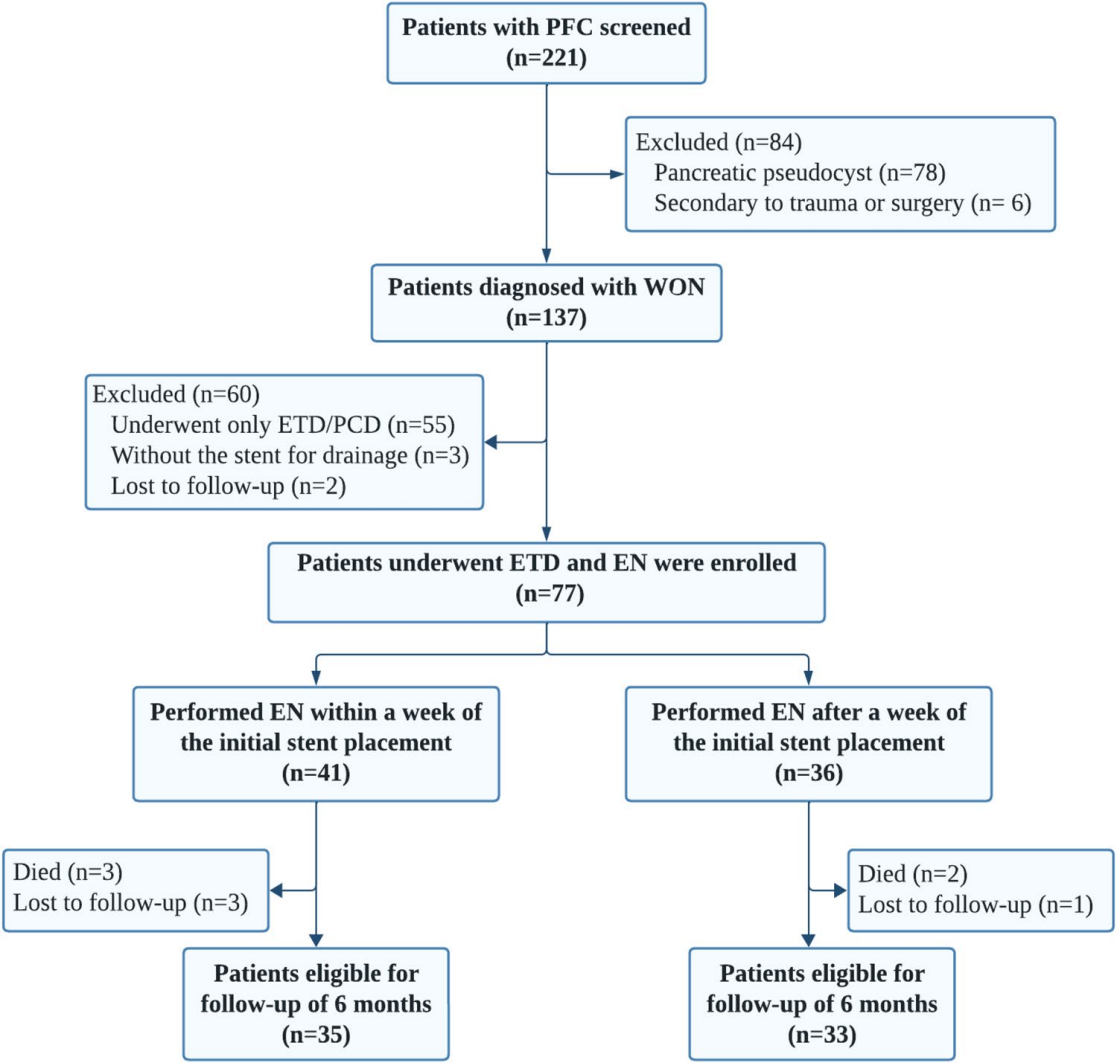
Trial profile.

A total of 221 patients with pancreatic or peripancreatic fluid collection at our single center were screened between Dec 2017 and Jun 2022. As shown in Fig. 3, among the 137 confirmed cases of WON, 40.1% of patients can be treated by ETD or PCD alone, and only 77 patients (56.2%) performed EN afterwards. All qualified patients were divided into two groups: early necrosectomy (41 patients) and postponed necrosectomy (36 patients). Before the initial necrosectomy, 73 patients (73/77, 94.8%) had received endoscopic transluminal drainage (ETD), and 26 patients (26/77, 33.8%) had received percutaneous catheter drainage (PCD) at our center.

The median age was 45 years (IQR 24), and 39% were female. Their body mass index (BMI) was 23.47 ± 3.51 kg/m^2^ on average. A total of 13.0% of patients had previously experienced episodes of acute pancreatitis. There were 12 former smokers and 16 drinkers. The underlying aetiology for pancreatitis included gallstones (37, 48.1%), high triglycerides (22, 28.6%), alcohol (8, 10.4%), idiopathic (6, 7.8%) and iatrogenic (4, 5.2%). The median major axis of the WON was 11.0 cm (IQR 4.3). The median CT severity index was 5.0 (IQR 2.0), and the bedside index of severity in acute pancreatitis was 2.0 (IQR 1.0). The median serum white blood cell count was 8.8 (IQR 6.0) × 10^9^/L, and C-reactive protein was 67.5 (IQR 95.3) mg/L at baseline. The culture of blood and cystic fluid information were available in 81.8% and 80.5% of enrolled patients, respectively. The overall positive rates of cystic fluid and blood culture were 96.8% and 21.0%, respectively.

The characteristics of the patients at baseline were equally distributed between the groups (Table 1). The following characteristics between the two groups did not differ significantly (P > 0.05): (1) Patient demographics: sex, age, BMI, smoking and drinking histories, history of AP, and pancreatitis aetiology; (2) The severity of illness: CTSI, BISAP, maximum size in cross section of WON, WBC and CRP at baseline; and (3) Preoperative relevant data: previous invasive intervention (ETD/PCD), the culture of blood and cystic fluid.

**Table 1:** Baseline characteristics. *BMI* body mass index, *CTSI* CT severity index, *BISAP* Bedside index of severity of acute pancreatitis, *WBC* White blood cell count, *CRP* C-reactive protein; the availability of secretion and blood culture was 81.8% and 80.5%, respectively.

	All patients (n = 77)	Early EN (n = 41)	Postponed EN (n = 36)	Z/χ^2^	P value
Age (years)	45.0 (24.0)	48.0 (21.0)	50.0 (32.0)	− 0.087	0.923
Sex				0.208	0.648
Female	30, 39.0%	15, 36.6%	15, 41.7%		
Male	47, 61.0%	26, 63.4%	21, 58.3%		
BMI (kg/m^2^)	23.47 ± 3.51	23.34 ± 4.01	23.38 ± 2.80	− 0.529	0.956
Previous history					
Smoking	12, 15.6%	7, 17.1%	5, 13.9%	0.148	0.701
Drinking	16, 20.8%	9, 22.0%	7, 19.4%	0.073	0.787
Acute pancreatitis	10, 13.0%	6, 14.6%	4, 11.1%	0.211	0.742
Pancreatitis aetiology			0.751	1.912	0.751
Gallstone	37, 48.1%	21, 51.2%	16, 44.4%		
High triglycerides	22, 28.6%	11, 26.8%	11, 30.6%		
Alcohol	8, 10.4%	5, 12.2%	3, 8.3%		
Idiopathic	6, 7.8%	3, 7.3%	3, 8.3%		
Iatrogenic	4, 5.2%	1, 2.4%	3, 8.3%		
Maximum size of WON	11.0 (4.3)	10.80 (4.80)	11.10 (3.93)	− 0.255	0.864
CTSI	5.0 (2.0)	4.0 (2.0)	5.5 (2.0)	− 0.289	0.772
BISAP	2.0 (1.0)	2.0 (1.0)	2.0 (1.0)	− 1.273	0.203
WBC (*10^9^)	8.8 (6.0)	8.9 (6.0)	8.7 (6.2)	− 0.562	0.574
CRP (mg/L)	67.5 (95.3)	67.0 (102.0)	70.9 (92.3)	− 0.143	0.886
Preoperative interventions					
PCD	26, 33.8%	16, 39.0%	10, 27.8%	1.084	0.298
ETD	73, 94.8%	37, 90.2%	36, 100%	3.705	0.054
Positive culture of cystic fluid	61, 96.8%	35, 97.2%	26, 96.3%	2.754	0.097
Positive culture of blood	13, 21.0%	7, 20.6%	6, 21.4%	0.007	0.936

### Procedural details and related complications

The average time from the stent placement and the necrosectomy of the two groups was 3 and 10 days. The procedural details are listed in Table 2. In terms of the entry route of endoscopy, in the early necrosectomy group, 35 patients (85.4%) underwent a transgastric approach, four patients (9.8%) had the percutaneous approach and two patients (4.9%) had a combined approach. In the postponed necrosectomy group, the rates of transgastric, percutaneous and combined approaches were 80.6%, 8.3% and 11.1%, respectively. In terms of stent types, the proportions of plastic stent and metal stent usage were 14.6% (6/41) and 85.4% (35/41) respectively in the early group, while in the postponed group, there were 25.0% (9/36) and 75.0% (27/36) respectively. The difference between the two groups was not statistically significant (χ^2^ = 1.313, P = 0.252). Finally, in our study, the necrosectomy tools used included snares, baskets and rat-toothed forceps.

**Table 2:** Procedural details and clinical outcomes.

	Early EN (n = 41)	Postponed EN (n = 36)	Z/χ^2^	P value
Stent type			1.313	0.252
Plastic stent	6, 14.6%	9, 25.0%		
Metal stent	35, 85.4%	27, 75.0%		
Entry route of endoscopy			2.673	0.215
Transgastric	35, 85.4%	29, 80.6%		
Percutaneous	4, 9.8%	3, 8.3%		
Combined	2, 4.9%	4, 11.1%		
Treatment success	28, 73.7%	26, 74.3%	0.003	0.953
Mortality	3, 7.3%	2, 5.6%	0.098	1.000
Additional interventions after EN	11, 26.8%	3, 8.3%	4.408	0.036*
ETD	2, 4.9%	0		
PCD	6, 14.6%	3, 8.3%		
Surgery	3, 7.3%	1, 2.8%		
Length of hospitalization (days)	49.0 (47.0)	38.0 (34.0)	− 0.960	0.337
Total number of EN for WON resolution	2.51 ± 1.49	1.89 ± 1.06	− 1.846	0.065
Follow-up examination				
Loss to follow-up	3, 7.9%	1, 2.9%	–	–
Recurrence	7, 20.0%	7, 21.2%	0.015	0.902
Endoscopic reintervention	4, 11.4%	5, 15.2%	0.205	0.730
Long-term adverse events	7, 20.0%	8, 24.2%	0.178	0.673
Pancreatic fistula	3, 8.6%	3, 9.1%	0.006	1.000
Chronic pancreatitis	1, 2.9%	3, 9.1%	1.192	0.349
Pancreatic endocrine insufficiency	3, 8.6%	2, 6.1%	0.157	1.000
Left portal hypertension	2, 5.7%	2 ,6.1%	0.004	1.000

**Figure 4:**
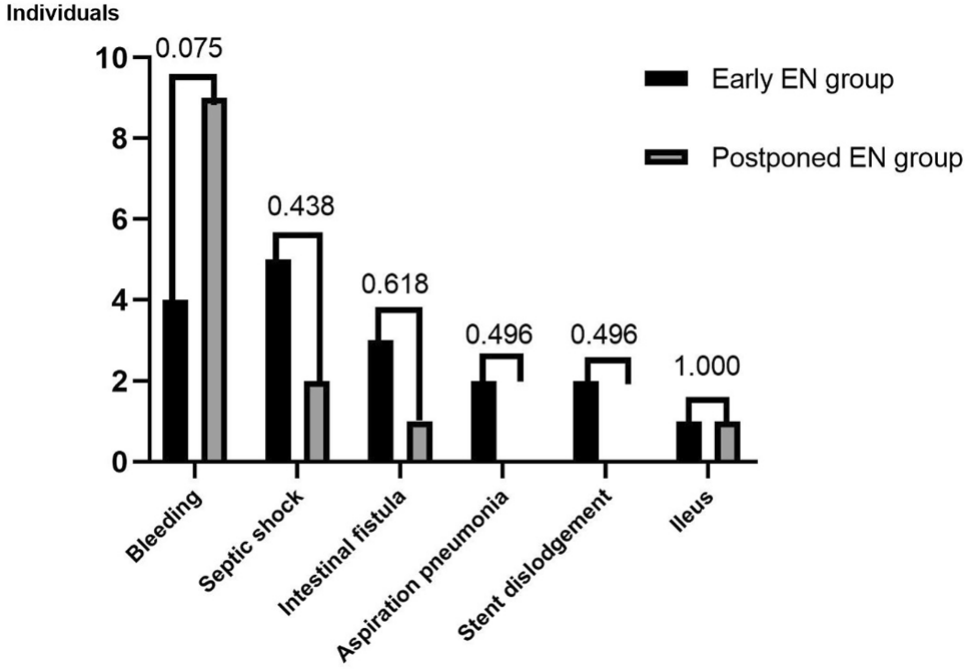
Procedure-related complications.

The procedure-related complications are listed in Fig. 4. The incidence of bleeding was 9.8% in the early necrosectomy group, compared to 25.0% in the postponed necrosectomy group, but the difference between groups was not statistically significant (χ^2^ = 3.174, P = 0.075). Among the four patients with bleeding in the early group, they all had metal stents placed. One experienced active bleeding from the cyst wall vessel during necrotic tissue removal, and another during gastrocystic access dilation, both effectively managed by thermal coagulation with forceps. The remaining two postoperative bleeding cases inside the cavity were successfully controlled by using thermal coagulation with forceps combined with 1:10,000 epinephrine irrigation and surgery, respectively. Among the nine patients in the postponed group, there were three cases using plastic stents and six cases using metal stents. Three experienced cyst wall vessel bleeding during necrotic tissue removal, which was effectively controlled by thermal forceps electrocoagulation or haemostatic powder spraying. Two patients encountered active bleeding during the dilation of the gastrocystic access and used thermal coagulation with forceps. Four patients experienced post-procedural bleeding, with specific outcomes as follows: one gastrointestinal bleeding case underwent emergency angiography and artery embolization; one abdominal bleeding case underwent angiography but no causative vessel was found, and eventually died due to refusal for surgery; one intracystic bleeding case was effectively treated with conservative management; and one intracystic bleeding case had a deteriorating condition with complications including septic shock and gastrointestinal perforation, eventually resulting in death. From the perspective of bleeding rates with different stent types, the incidence of bleeding with plastic stents and metal stents was 20.0% (3/15) and 16.1% (10/62) respectively, with no statistically significant difference between the two stents (χ^2^ = 0.129, P = 0.710).

There were no statistically significant differences observed in the occurrence of other complications, including septic shock (12.2% in the early necrosectomy group and 5.6% in the postponed necrosectomy group; χ^2^ = 1.022, P = 0.438), intestinal fistula (7.3% and 2.8%, respectively; χ^2^ = 0.802, P = 0.618), aspiration pneumonia (4.9% and 0, respectively; χ^2^ = 1.803, P = 0.496), stent dislodgement (4.9% and 0, respectively; χ^2^ = 1.803, P = 0.496), ileus (2.4% and 2.8%, respectively; χ^2^ = 0.009, P = 1.000). In short, the overall complication rate between the two groups was not significantly different (29.3% in the early group vs. 30.6% in the postponed group; χ^2^ = 0.409, P = 0.522).

### Clinical outcomes

The clinical outcomes are listed in Table 2. First, the mortality of the early and postponed necrosectomy groups were 7.3% and 5.6%, respectively, and the results were similar (χ^2^ = 0.098, P = 1.000). A total of five patients in our cohort died after active treatment, and their main causes of death were as follows: two patients died of septic shock, one patient died of progressive cyst haemorrhage, and the others died of intraperitoneal haemorrhage and multiple organ failure. There was no significant difference between the early necrosectomy group and the postponed necrosectomy group in terms of the rate of complete WON resolution within six months of follow-up (73.7% vs. 74.3%, respectively; χ^2^ = 0.003, P = 0.953).

Second, 14 patients received additional invasive interventions after EN, including nine patients with PCD, and the average number was 1.6 (1–3), four patients with surgery and two patients with ETD for complete WON resolution. The additional intervention rate after the initial EN differed significantly between the two groups (26.8% in the early necrosectomy group vs. 8.3% in the postponed necrosectomy group; χ^2^ = 4.408, P = 0.036). The median hospital stay of the early necrosectomy group was 49.0 (47.0), and that of the postponed necrosectomy group was 38.0 (34.0) (Z = − 1.031, P = 0.302).

Finally, regarding the overall frequency of necrosectomy, 29 (29/77, 37.7%) patients achieved a resolution of WON with a single endoscopic necrosectomy. Although the postponed necrosectomy group had fewer session necrosectomy than the early group, this difference was not statistically significant (1.89 vs. 2.51, respectively; Z = − 1.846, P = 0.065).

### Postdischarge follow-up

All available patients were discharged after controlling infection and followed for six months. The rate of loss to follow-up was 5.6% (4/72). Seven patients (7/35, 20.0%) in the early EN group were readmitted due to disease recurrence, of whom four patients (4/35, 11.4%) underwent endoscopic reintervention. The readmission rate of the postponed EN group was 21.2% (7/33), of which five patients (5/33, 15.2%) underwent endoscopic reintervention. No differences were observed in the readmission and endoscopic reintervention rates between the two groups (χ^2^ = 0.031, P = 0.860). Additionally, seven patients (7/35, 20.0%) in the early EN group experienced long-term adverse events (Table 2), which included three pancreatic fistula cases, three pancreatic endocrine insufficiency cases, two left portal hypertension cases, and one chronic pancreatitis case. In the postponed EN group, eight patients (8/33, 24.2%) experienced long-term AEs, including three with pancreatic fistula, three with chronic pancreatitis, two with pancreatic endocrine insufficiency, and two with left portal hypertension. The overall rate of long-term complications did not significantly differ between the two groups (χ^2^ = 0.178, P = 0.673). Similarly, there were no significant differences observed in the incidence of the following specific complications: pancreatic fistula (8.6% in the early necrosectomy group and 9.1% in the postponed necrosectomy group; χ^2^ = 0.006, P = 1.000), chronic pancreatitis (2.9% and 9.1%, respectively; χ^2^ = 1.192, P = 0.349), pancreatic endocrine insufficiency (8.6% and 6.1, respectively; χ^2^ = 0.157, P = 1.000) and left portal hypertension (5.7% and 6.1%, respectively; χ^2^ = 0.004, P = 1.000).

## Discussion

Many endoscopists may prefer to choose a step-up strategy to avoid unnecessary interventions. Van Brunschot et al. found that 51% of surgical patients were successfully treated with catheter drainage only and more than 40% of patients in the endoscopy group were also successfully treated with endoscopic drainage only without additional necrosectomy^8^. However, considering the large size of WON, the small diameter of the stent or the higher proportion of solid debris in the cavity, EN has improved clinical outcomes for the management of WON^16^. In 2000, Seifert et al. described the first successful case of endoscopic necrosectomy for WON to remove necrotic tissue effectively^17^. A retrospective study comparing direct endoscopic necrosectomy with ETD for WON found that without a concurrent change in the frequency of endoscopic interventions, the incidence of complications, or hospital stays, EN can achieve a higher rate of successful WON resolution^10^. Although EN is effective in the treatment of WON, PCD or ETD as the first step allows 35–50% of patients to avoid endoscopic percutaneous or transgastric necrosectomy^8^, which is also consistent with our findings. Our research indicates that if there is no strong indication mentioned above, delaying necrosectomy has the potential to avoid unnecessary interventions. However, the latest prospective study indicates that patients who received upfront endoscopic necrosectomy rather than as a step-up measure have less number of reinterventions required to achieve treatment success^18^. The optimal timing of necrosectomy is still controversial, and futher high-quality studies with larger sample sizes are needed to clarify this in the future.

Currently, to mobilize necrotic debris early, an increasing number of endoscopists prefer to carry out EN with initial stent placement. Yan et al. compared the clinical outcomes for ETD followed by immediate or delayed DEN in 271 patients with WON. The results indicated that the clinical success rates for resolution were 91.3% and 86.1% in the immediate and delayed DEN groups, respectively (P = 0.3), and the mean number of necrosectomy for clinical success in the immediate group was considerably lower than that in the delayed group^11^. Conversely, our study suggests that without compromising the incidence of procedure-related adverse events and the clinical success rate, postponing necrosectomy beyond one week may reduce the risk of subsequent reintervention. Recently, a retrospective comparative cohort analysis revealed that patients who underwent delayed DEN had shorter hospital stays and fewer necrosectomy sessions than those who underwent immediate DEN^19^. Consequently, delaying EN for one week after drainage with stents can reduce the additional reintervention rate (P = 0.036) and may also lower the need for repeated necrosectomy (P = 0.065).

However, we also found that the rate of bleeding in the postponed group was significantly higher than that of the early group. Although our study does not allow for a direct investigation to explain such a high incidence of bleeding (25.0%). There are several possible explanations for the high rate of bleeding in the postponed group. First, there is no denying that this may be related to disease progression and it is random. Second, it may be related to the longer reservation time of the stent. 76.9% (10/13) of bleeding patients received LAMS placement in our study. Patients in the postponed group reserved LAMS for 26.8 days on average, while those in the early group usually removed LAMS during the first EN and placed the plastic stent if necessary. Fugazza et al. found that the incidence of LAMS stent-related adverse events was 24.3%, including bleeding, stent displacement, infection, stent blockage, stent embedding, and pyloric obstruction, among which 43.0% of adverse events occurred within 14 days of LAMS placement, and bleeding and infection were most common early complications. Similarly, Lang et al. also found that the rate of delayed bleeding for LAMS was as high as 17%, occurring on average 9.5 days after stent placement^20^. We hypothesized that as WON resolves, LAMS remains in place due to its bi-flanged design. On the one hand, it allows more entry of gastric acid into the cavity, and the low PH fluid may irritate exposed intracavitary vessels and promote bleeding. On the other hand, the flange of the stent continuously stimulates the WON wall, promoting the formation of false aneurysms^21^. Nevertheless, the endoscopic step-up approaching using LAMS is safe and effective, with technical and clinical success rates reaching 99% and 74.6%, respectively^22^. Most bleeding can be effectively controlled with conservative treatment, while some require DSA, endoscopy or surgery. Recently, Koga first reported successful endoscopic haemostasis by PuraStat, a novel technique using self-assembling peptide gel, which can facilitate the detection of bleeding sites^23^. Therefore, PuraStat is expected to be a more effective and safer option for EN-related bleeding. We should pay attention to avoid macroscopic blood vessels during debridement, and it is not recommended to remove too much necrosis attached to the wall of large vessels during the procedure. Additionally, perforation is the second most common complication, and the majority can be managed conservatively^24^.

Our findings do not confirm the hypothesis that performing EN early after ETD leads to better patient outcomes with a lower reintervention rate. These findings differ from several previous studies^10,11^. A recent retrospective comparative cohort analysis involving 80 patients with WON assessed outcomes after immediate EN compared with delayed EN and showed that patients in the early-necrosectomy group required longer hospital stays and more necrosectomy sessions than patients in the delayed-necrosectomy group^19^. Our study also found that the mean number of necrosectomy was lower in the postponed group than in the early group (1.89 vs. 2.51, respectively). Conversely, Yan et al. demonstrated that performing EN at the time of stent placement resulted in a lower number of necrosectomy sessions for WON resolution^11^. Additional interventions, including ETD, PCD and surgery, were necessary in 26.8% of patients treated with early necrosectomy but in only three patients (8.3%) in the postponed necrosectomy group. We speculate that patients in the early group may have more subsequent index necrosectomy due to disease progression and increased necrotic matter, but patients in the postponed group had enough time to drain the necrosis and the cavity was more mature.

Our study directly evaluates the effect of performing necrosectomy one week after the initial stent placement on the clinical success rate and long-term outcomes in cases of similar disease severity and baseline data. The strengths of our study are the large number of patients enrolled and the comprehensive evaluation of the clinical efficacy, procedure-related complications and long-term prognosis of the two groups. However, the lack of randomization in our study is a major concern. Since the choice to perform an early necrosectomy could have been based on WON of more complexity, possibly requiring a reintervention thereafter. In addition, some other limitations of our study are inherent to retrospective studies, which included incomplete clinical data, the effect of unobserved bias and confounding factors and variability of the endoscopist technique. We look forward to larger-scale randomized controlled trials in the future to further validate these findings.

In conclusion, this study showed that postponing EN may avoid additional interventions for infected WON. In terms of clinical success rate, total count of necrosectomy procedures, procedure-related complications, length of hospitalization and prognosis, our study did not demonstrate that early necrosectomy is superior to postponed necrosectomy.

## Data Availability

Corresponding authors can be contacted for relevant data upon reasonable request.
